# Effectiveness of Probiotic Use in Alleviating Symptoms of Irritable Bowel Syndrome: A Systematic Review

**DOI:** 10.7759/cureus.58306

**Published:** 2024-04-15

**Authors:** Lotanna Umeano, Sadaf Iftikhar, Sarah F Alhaddad, Christian N Paulsingh, Muhammad Faisal Riaz, Gourav Garg, Lubna Mohammed

**Affiliations:** 1 Internal Medicine, California Institute of Behavioral Neurosciences & Psychology, Fairfield, USA; 2 Pediatric, California Institute of Behavioral Neurosciences & Psychology, Fairfield, USA; 3 Pathology, St. George's University School of Medicine, St. George's, GRD; 4 Internal Medicine, Rawalpindi Medical University, Rawalpindi, PAK; 5 Orthopedics, King's Mill Hospital, Sutton-in-Ashfield, GBR

**Keywords:** gut, ibs, irritable bowel syndrome, gut microbiota, probiotics

## Abstract

Irritable bowel syndrome (IBS) is a common functional gastrointestinal (GI) condition, and changes in the gut microbiota's composition contribute to the development of symptoms. Although the precise mechanisms of probiotic use in the human body are not fully understood, probiotic supplements are believed to reduce symptoms, such as abdominal pain, by regulating neurotransmitters and receptors associated with pain modulation in IBS patients compared to placebo by altering the gut flora. This systematic review aimed to assess the most current randomized controlled trials (RCTs) on how probiotic supplementation affects the symptoms in people with IBS. The effects of probiotic supplements on IBS symptoms were studied in RCTs published between January 2018 and June 2023. After a search through PubMed and Google Scholar using the keywords probiotics, gut microbiota, irritable bowel syndrome, and IBS; eight articles matched the inclusion criteria and were reviewed. Four trials used a multistrain probiotic, whereas the remaining four trials examined the effects of a monostrain supplement. All eight trials came to the same conclusion: Probiotic treatment may significantly reduce symptoms.

## Introduction and background

Irritable bowel syndrome (IBS) is a functional gastrointestinal (GI) disorder that substantially impacts quality of life and social functioning. Between 5% and 10% of the general population are affected by this disorder [1]. IBS is characterized by recurring stomach pain coupled with irregular stools in either form or frequency. Some symptoms include bloating, flatulence, abdominal pain, or discomfort triggered by a change in bowel habits (diarrhea, constipation, or a combination of the two). IBS is thought to be multifaceted, with genetic, psychological, and environmental variables all playing a role. However, the pathophysiology of IBS needs to be better understood. Among the hypothesized processes are dysbiosis in the gut microbiota, immunological activation, aberrant entero-endocrine signaling, altered GI motility, and disruptions in the integrity of the epithelial barrier [2].

The Rome criteria, the symptom-based diagnostic criteria for IBS and other functional GI diseases (FGIDs), are used to diagnose IBS when other severe GI illnesses are ruled out [3]. The Rome IV criteria were developed by consensus among experts in functional GI disorders. The criteria described in Table 1 consist of abdominal pain associated with an alteration in either stool form or frequency occurring for at least six months [1]. Patients are subgrouped according to predominant stool pattern using the Bristol Stool Form Scale: IBS with diarrhea (IBS-D), IBS with constipation (IBS-C), IBS with mixed stool pattern (IBS-M), and IBS unclassified (IBS-U).

**Table 1:** IBS subclassifications IBS: Irritable bowel syndrome

		Diagnostic criteria
IBS		Recurrent abdominal pain, on average for at least one day per week in the past three months, associated with two or more of the following: related to defecation, a change in stool frequency, or a change in stool form; the criteria must be fulfilled for the past three months, with symptom onset at least six months before diagnosis
IBS with constipation		≥25% of bowel movements of Bristol Stool Form types 1 or 2, and <25% of Bristol Stool Form types 6 or 7
IBS with diarrhea		≥25% of bowel movements of Bristol Stool Form types 6 or 7, and <25% of Bristol Stool Form types 1 or 2
IBS with mixed stool pattern		≥25% of bowel movements of Bristol Stool Form types 1 or 2, and ≥25% of bowel movements of Bristol Stool Form types 6 or 7
IBS unclassified	Patients who meet the criteria for IBS but do not fall into one of the other three subgroups according to the Bristol Stool Form type	

The medical treatment of IBS typically focuses on addressing the specific symptom that the patient is experiencing the most. In addition, it has been demonstrated that a diet low in fermentable oligosaccharides, disaccharides, monosaccharides, and polyols (FODMAPs) reduces the symptoms of IBS, and this diet is currently advised [4]. Antispasmodics, bulking agents, psychotropics, and serotonin (5-hydroxytryptamine (5-HT) receptor antagonists are also recommended for treating IBS. The varied etiology of the disease may be to blame for the fact that, in most cases, these medications have not been successful in alleviating symptoms. However, not being fatal, IBS causes uncomfortable symptoms for those who have it. It is also associated with increased rates of anxiety and depression as well as economic issues, all of which can lead to a severe decline in the quality of life (QoL). Alternative methods to alleviate symptoms and enhance the QoL for those affected are desperately needed because neither pharmacological treatment nor dietary adjustments typically completely eradicate symptoms [5].

The gut-associated microbiota is commonly known as a "super organ" due to its multifaceted bodily functions. Its vital roles include aiding digestion and nutrient absorption, boosting immunity, shaping the intestinal epithelial barrier, and influencing the microbiota-gut-brain axis [6]. The intricate relationships between the GI tract, the central, peripheral, and autonomic nervous systems, and neuroendocrine pathways are called the "brain-gut axis." Brain-to-gut linkages are demonstrated by the development of new GI symptoms in individuals with preexisting psychological illnesses, and these interconnected pathways are expected to play a significant role in the pathophysiology of functional GI diseases. Those who already report GI problems developing anxiety or depression de novo support the existence of gut-brain linkages. This simultaneous brain-to-gut and gut-to-brain activity highlights the importance of bidirectional brain-gut axis interactions in IBS and other functional GI diseases [7].

A hypothesis derived from clinical observations of symptoms appearing after infection and widely known as postinfectious IBS is indicated as a possible contributor to IBS [8,9]. It is important to note that small intestinal bacterial overgrowth (SIBO) can cause symptoms similar to IBS, mainly bloating after eating. Studies have shown differences in the gut microbiome profile of IBS patients compared to healthy individuals, and specific symptoms and severity of the illness have been linked to particular gut microbial compositions [10-14], and specific symptoms and illness severity have been linked to distinct gut microbial compositions [15,16].

Probiotics are live microorganisms that provide health benefits to the host when taken in adequate amounts. This definition was established by the Food and Agriculture Organization (FAO) and the World Health Organization (WHO) in 2001 [17]. Elie Metchnikoff, a Russian Nobel Laureate who noticed that eating fermented foods containing lactic acid bacteria positively impacted human health, originally proposed the idea of probiotics in 1908. He ingested sour milk daily and is credited with coining the term "yogurt" based on his notion that lactic acid could lengthen life. Since then, the effects of probiotics have been extensively researched in a wide range of illnesses, and they are now considered a potential treatment or preventative measure for many GI ailments [18,19]. Probiotics' potential to reverse dysbiosis (qualitative and quantitative changes in the microbiota) or stabilize the host microbiota is the justification for their use in managing IBS. The method of probiotics' activity in IBS needs to be clarified.

The influence of gut microbiota on health encompasses several vital mechanisms. These include microbial competition and the inhibition of pathogens, facilitated by producing substances like bacteriocins, short-chain fatty acids (SCFAs), and biosurfactants. It also enhances the gut barrier's integrity by regulating immune responses, bolstering the mucus layer, and fortifying tight junction proteins. Furthermore, gut microbiota exhibits anti-inflammatory effects by suppressing proinflammatory cytokines and boosting gut immunity by stimulating secretory IgA production. This complex interplay also extends to communication with the brain, highlighting the profound impact of gut health on overall well-being [20,21].

Research in both animal and human subjects indicates that distinct strains of probiotics may alleviate abdominal pain and diminish visceral hypersensitivity. This is achieved through regulating neurotransmitters and receptors associated with pain modulation, including the opioid and cannabinoid receptors. These findings highlight the potential therapeutic impact of probiotics on GI discomfort and hypersensitivity in various populations [22]. However, identifying specific bacterial strains or probiotic supplements positively affecting IBS symptoms can lead to more effective treatment approaches. The theory that probiotic supplements alleviate IBS symptoms by changing the gut microbiota or its metabolic pathways still needs mechanistic evidence [5]. This systematic review aims to evaluate the most current randomized controlled trials (RCTs) and examine the impact of probiotic supplementation on IBS patients' symptoms.

This review paper is based on an understanding of the crucial role that gut microbiota plays in IBS. Existing research has robustly demonstrated that alterations in the composition and function of gut microbiota are key contributors to developing and progressing IBS. The review's unique focus on recent RCTs published within the last five years ensures it captures the latest advancements in probiotic research. This allows for identifying potentially more effective probiotic strains or formulations, providing clinicians and researchers with up-to-date, evidence-based insights for managing IBS. Ultimately, the review seeks to enhance therapeutic approaches and refine strategies for individuals grappling with this challenging GI condition.

## Review

Method

For this systematic review, the Preferred Reporting for Systematic Reviews and Meta-Analyses (PRISMA) checklist and flowchart were used [23].

Search strategy and criteria for inclusion

After conducting a thorough search on PubMed and Google Scholar, we found 118 studies. However, only eight studies met the eligibility criteria and were included in the review. Table 2 shows the databases used and the identified numbers of papers for each database, using the keywords probiotics, gut microbiota, irritable bowel syndrome, and IBS. Patients with IBS who met the Rome III and Rome IV criteria were included in these double-blind, placebo-controlled, randomized trials. These studies were published within the previous five years between January 2018 and June 2023. We employed specified inclusion and exclusion criteria listed in Table 3. Each study examined how patients who got probiotics or a placebo responded to the IBS symptoms.

**Table 2:** Keywords/strategies used and the number of identified papers

Search strategy	Database used/date searched	Number of research papers identified
Probiotics OR Gut Microbiota ("Probiotics/administration and dosage" [Majr] OR "Probiotics/pharmacokinetics" [Majr] OR "Probiotics/pharmacology" [Majr] OR "Probiotics/therapeutic use" [Majr]) AND Irritable bowel syndrome OR IBS ("Irritable Bowel Syndrome/classification" [Mesh] OR "Irritable Bowel Syndrome/diagnosis" [Mesh] OR "Irritable Bowel Syndrome/drug therapy" [Mesh] OR "Irritable Bowel Syndrome/microbiology" [Mesh] OR "Irritable Bowel Syndrome/physiopathology" [Mesh] OR "Irritable Bowel Syndrome/prevention and control" [Mesh] OR "Irritable Bowel Syndrome/rehabilitation" [Mesh] OR "Irritable Bowel Syndrome/therapy" [Mesh])	PubMed 21/6/2023	98
(Probiotics in irritable bowel syndrome) AND (("2018"[Date - Entry] : "2023"[Date - Entry]))	PubMed 21/6/2023	21
Probiotics + Irritable bowel syndrome+ Clinical trial	Google Scholar 21/6/2023	9

**Table 3:** Inclusion and exclusion criteria IBS: Irritable bowel syndrome; RCT: randomized controlled trial

Inclusion	Exclusion
IBS patients	Healthy non-IBS patients
Human studies	Animal studies
Studies in adults	Studies in children
RCTs	Studies without RCT methodology
Double or triple-blinded studies	Single-blinded or partially-blinded studies
Studies published in the last five years	Studies older than five years
IBS diagnosis with Rome III or Rome IV criteria	IBS diagnosis with Rome II or Manning criteria
Studies looking at a change in IBS symptoms as the primary outcome	Studies not looking at a change in IBS symptoms as primary outcome
Studies looking solemnly at probiotics in an intervention group	Studies looking at probiotics in conjunction with other IBS therapies in the same intervention group

Results 

**Figure 1:**
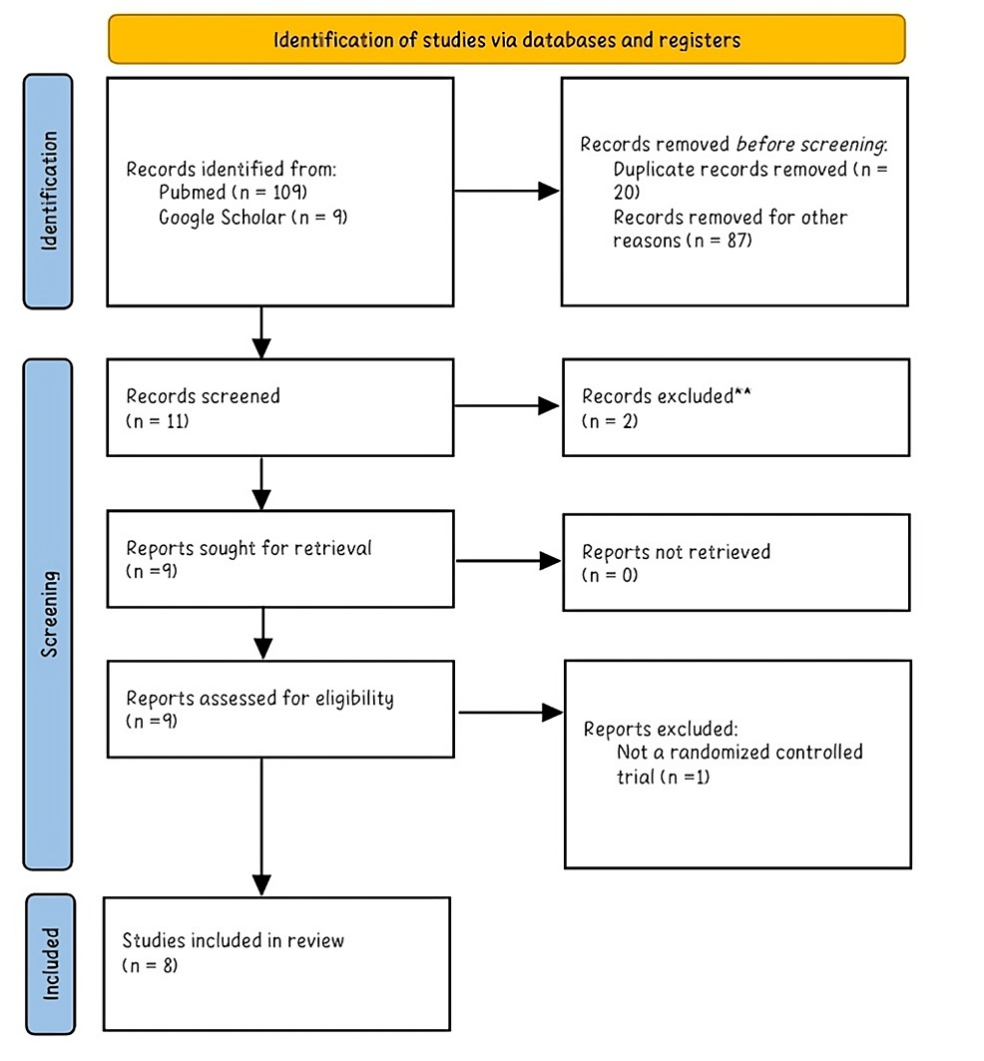
Search results depicted in the PRISMA flowchart 2020 PRISMA: Preferred Reporting for Systematic Reviews and Meta-Analyses

Based on the search, a total of 118 studies were reviewed, and 107 were excluded; the excluded studies were either duplicates, conducted in children, non-IBS patients, or healthy individuals, or they evaluated the effect of combination therapy on IBS symptoms. We have confidently included eight studies in our research through meticulous data analysis. Our selection process, excluding three studies based on their abstract or full-text review, assures the accuracy and reliability of our findings. Eight studies were used in the systematic review, which evaluated the effect of probiotic supplementation on IBS symptoms. All studies had a randomized, double-blinded, placebo-controlled trial methodology and included IBS patients diagnosed according to the Rome III criteria. The studies were conducted in Europe or Asia; a summary of the included studies is in Table 4. The PRISMA flow diagram is depicted in Figure 1.

**Table 4:** Overview of the eight studies included in the systematic review N: Sample size; CFU: colony-forming units; IBS-SSS: Irritable Bowel Syndrome Severity Scoring System; IBS-GIS: Irritable Bowel Syndrome Global Improvement Scale; APS-NRS: Abdominal Pain Severity-Numeric Rating Scale; HADS: Hospital Anxiety and Depression Scale; QoL: quality of life; IBS-C: irritable bowel syndrome with predominately constipation; IBS-D: irritable bowel syndrome with predominant diarrhea; IBS-M: irritable bowel syndrome with a mixture of both diarrhea and constipation; IBS-U:  irritable bowel syndrome uncategorized; SF-36: Short Form Health Survey questionnaire

First author, year of publication, country	N	Probiotic strains (amount)	Probiotic form	Dose	IBS subtype	Study duration	Symptom evaluation
Monostrained probiotics							
Gupta, 2021, India [24]	40	*Bacillus coagulans LBSC [DSM17654*] 6 billion/d	Powder	Thrice daily	Not specified	80 days 11 weeks	IBS-SSS Bristol Stool Form Scale
Lewis, 2020, Canada [25]	251	Lactobacillus paracasei HA-196 (L. paracasei) and Bifidobacterium longum R0175 (B. longum)	Capsule	Daily	IBS-D, IBS-C IBS-M (mixed pattern)	10 weeks	IBS-SSS IBS-QoL HADS, SF-36
Madempudi, 2019, India [26]	153	B. coagulans Unique IS2 2 billion per CFU	Capsule	Daily	Not specified	8weeks	Mean complete spontaneous bowel movement (CSBM)
Martoni, 2020, Switzerland [27]	336	Lactobacillus acidophilus DDS-1 (1 × 10^10^ CFU/day) Bifidobacterium animalis subsp. lactis UABla-12 (1 × 10^10^ CFU/day)	Capsule	Daily	Not specified	6weeks	APS-NRS IBS-SSS Bristol Stool Scale (BSS) IBS-QoL
Multistrained probiotics							
Ishaque, 2018, Bangladesh [28]	400	Bacillus subtilis PXN 21, Bifidobacterium spp. (B. bifidum PXN 23, B. breve PXN 25, B. infantis PXN 27, B. longum PXN 30), Lactobacillus spp. (L. acidophilus PXN 35, L. delbrueckii spp. Bulgaricus PXN39, L. casei PXN 37, L. plantarum PXN 47, L. rhamnosus PXN 54, L. helveticus PXN 45, L. salivarius PXN 57), Lactococcus lactis PXN 63, and Streptococcus thermophilus PXN 66]	Capsule	Twice daily	IBS-D	16 weeks	IBS-SSS IBS-QoL
Oh, 2019, Vietnam [29]	50	*Lactobacillus paracasei, Lactobacillus salivarius, and Lactobacillus plantarum 1 × 10^9^ CFU*	Capsule	Daily	IBS-D, IBS-M, IBS-U	4 weeks	Subject global assessment (SGA), visual analog score (VAS)
Sadrin, 2020, France [30]	80	Lactobacillus acidophilus 5 × 10^9^ cfu/capsule	Capsule	Twice daily	Not specified	8 weeks	100 mm visual analog scale
Skrzydło-Radomańska, 2021, Poland [31]	48	Bifidobacterium breve, Bifidobacterium longum, Bifidobacterium bifidum, Bifidobacterium lactis, Lactobacillus rhamnosus, Lactobacillus paracasei, Lactobacillus acidophilus, Lactobacillus casei, Lactobacillus plantarum, Streptococcus thermophilus	Capsule	Daily	IBS-D	8 weeks	IBS-SSS scale, IBS-GIS Bristol Stool Scale

Main findings

Table 5 summarizes the main findings. The reported main findings are consistent among the eight studies in this review. All eight studies concluded that supplementation with probiotics in IBS patients may significantly improve symptoms compared to placebo.

**Table 5:** Overview of the findings and outcome metrics from the eight trials TNF: Tumor necrosis factor; IL: interleukin; HADS: Hospital Anxiety and Depression Scale; VAS: visual analog scale; SGA: subject global assessment; IBS-SSS: Irritable Bowel Syndrome Symptom Severity Score; IFN-γ: interferon-gamma

First author, year published, country	Primary outcome	Main findings, primary outcome	Secondary outcome	Main findings, secondary outcome
Gupta, 2021, India [24]	Bloating/cramping, abdominal pain, diarrhea, constipation, stomach rumbling, nausea, vomiting, headache, and anxiety	Significant improvement in primary outcome	Stool consistency	Significantly improved
Lewis, 2020, Canada [25]	Change in severity and frequency of abdominal pain, gastrointestinal and psychological symptoms	*L. paracasei *reported improvements in their bowel habits and stool consistency, *L. paracasei *and *B. longum* improved psychological well-being	Stool frequency and consistency, quality of life	Improvement of symptoms, emotional well-being, and social functioning
Madempudi, 2019, India [26]	Abdominal pain and discomfort, complete spontaneous bowel movements	Significant improvement and an increase in complete spontaneous bowel movement	Changes in pro and anti-inflammatory cytokines	The levels of pro-inflammatory cytokines (IL-6, IL-12, TNF-α, IFN-γ) and anti-inflammatory cytokine (IL-10) did not significantly change
Martoni, 2020, Switzerland [27]	Abdominal pain severity, abdominal distension, and bowel habits	Significant improvement in symptoms	Stool consistency, quality of life	Normalization of stool consistency and improved quality of life.
Ishaqe, 2018, Bangladesh [28]	Severity of abdominal pain in patients	Significant improvement	Number of bowel motions per day, quality of life	Reduction in bowel motion and markedly improved quality of life
Oh, 2019, Vietnam [29]	Overall IBS symptoms	Significant improvement in overall IBS symptoms	SGA scores and VAS scores	Significant improvements in SGA scores and reductions of VAS scores
Sadrin, 2020, France [30]	Abdominal pain	There was significant improvement in both groups, but no difference between them	Bloating, flatulence, and rumbling. Safety of mixture	Significant differences between groups were found for flatus scores at week four
Skrzydło-Radomańska, 2021, Poland [31]	Changes in symptom severity and improvement	Improved symptom severity significantly more significant reduction in the total IBS-SSS	Changes in stool consistency, number of bowel movements per day, severity of pain and flatulence, fecal urgency, and occurrence of adverse effects	Significant improvement in symptoms except for the severity of flatulence

Of the eight studies included, four examined the effects of a monostrain supplement, which contained just one strain of microorganisms [24-27]. In contrast, the remaining four trials examined the effects of multistrain supplements, which included combinations of two to 15 distinct bacteria [28-31]. Additionally, all studies reviewed in this article reported substantially reducing IBS symptoms. All of the multistrain probiotics used in the studies involve bacterial strains. However, different combinations of bacterial strains are used in each. Some bacterial strains and strain combinations are more prevalent than others. According to the vast majority of probiotics available on the market, *Bifidobacterium* and *Lactobacillus* are the two bacterial genera most frequently found among the probiotics delivered in the included research.

The frequency and amount of multistrain probiotic administration varied among trials. Two studies investigating a multistrain supplement gave probiotic tablets twice daily [28,30], while the other two studies used one supplement per day [27,29]. Additionally, there were variations in the frequency and amount of dosage used for the monostrain probiotics. One study used probiotic capsule supplementation three times a day and looked into a monostrain supplement; the other research only used one daily supplement. Probiotic supplementation dosages varied significantly between studies (see amount reported in Table 4). Hence, the effect of the frequency of supplementation may be less relevant than the amount of probiotics in each supplement/capsule, and the results of the frequency of supplementation cannot be concluded based on current data.

The duration of the various trials ranged from four to 16 weeks of intervention, while the study populations ranged in size from 40 to 400 people. Additionally, the methods used to assess the severity of the symptoms varied across the studies; some relied on medical assessments, while others used instruments like the standardized bowel disease questionnaire (SBDQ), the visual analog scale (VAS), and the IBS symptom severity score (IBS-SSS). The criteria for participant exclusion differed between research as well. Studies, however, excluded patients taking medication (such as antibiotics) or receiving additional IBS treatment.

Studies Evaluating the Effect of Monostrain Probiotics

Four of the included studies evaluated the effect of a monostrain probiotic on IBS symptoms [24-27]. Two of these studies used different strains of the same microorganisms in their probiotic supplement: *Bacillus coagulans* [24,26]. Two studies compared two different strains of bacteria with a placebo, administering the supplements in a ratio of 1:1:1 [25,27]. All four studies reported an improvement in the primary and secondary outcomes assessed.

Gupta et al. conducted a study that included 40 patients with IBS and 80 days of probiotic supplementation [24]. The probiotic supplement consisted of three daily tablets of the *Bacillus coagulans LBSC *(DSM17654). After the intervention, they reported a significant improvement in the intervention group compared to the placebo group in all primary outcomes: boating and cramping (p = 0.0148), relief from abdominal pain (p < 0.0001), improvement in diarrhea and constipation (p = 0.0027), improvement in stool consistency (p = 0.0002), and nausea and vomiting (p = 0.0031).

Lewis et al. conducted a trial that included 251 participants with IBS [25]. They studied two different strains, *Lactobacillus paracasei HA-196 (L. paracasei)* and *Bifidobacterium longum R0175 (B. longum)*. The probiotic was administered once daily for 10 weeks. The participants were split into three groups: *L. paracasei, B. longum,* or placebo. Despite no significant between-group differences, the IBS-SSS baseline significantly decreased in all three groups. IBS-SSS scores were considerably lower in the* L. paracasei* (30%),* B. longum* (22%), and placebo (31%) groups at week eight compared to baseline (all p = 0.001). Rescue medication (bisacodyl 5 mg tablets) was approved to treat extreme constipation. However, compared to the placebo group, both probiotic-supplemented groups reported taking less rescue medication. This difference was only shown to be statistically significant for the *L. paracasei* group (p = 0.05). In the* L. paracasei* group, the frequency of complete spontaneous and spontaneous bowel movements increased in people with IBS-C after eight weeks of supplementation. It was reduced in participants with IBS-D (p = 0.013).

Madempudi et al. studied the *B. coagulans* *Unique IS2* administered as a probiotic supplement in a daily capsule [26]. The study was an eight-week intervention that included 153 adults with IBS. This study evaluated how *B. coagulans Unique IS2 *supplementation affected persons with IBS regarding abdominal discomfort, complete spontaneous bowel movements (CSBMs), and illness severity. The mean baseline total severity symptoms score of* B. coagulans* group decreased (p < 0.0001) from 26.4 ± 2.54 to 10.6 ± 5.26. The mean baseline CSBM score of* B. coagulans Unique IS2* group was increased from 2.5 ± 1.54 to 4.0 ± 1.43. The mean score of baseline pain was reduced from 8.2 ± 1.37 to 3.4 ± 2.08 in *B. coagulans Unique IS2*-treated group. *B. coagulans* did not significantly alter the TNF, IL-6, IL-10, and IL-12 serum levels compared to placebo.

Martoni et al. conducted a trial that included 336 participants with IBS [27]. They studied two different strains, *Lactobacillus acidophilus DDS-1* and *Bifidobacterium animalis subsp. lactis UABla-12*. For six weeks, the probiotic was given orally once a day. Three groups of participants each received *L. acidophilus DDS-1, B. lactis UABla-12*, or a placebo. The primary outcome was the change in the Abdominal Pain Severity-Numeric Rating Scale (APS-NRS). Throughout the intervention, both probiotic groups significantly outperformed the placebo in terms of APS-NRS (DDS-1: 2.59 2.07, p = 0.001; UABla-12: 1.56 1.83, p = 0.001). IBS-SSS scores for the* L. acidophilus DDS-1* (133.4 95.19, p 0.001) and *B. lactis UABla-12* (104.5 96.08, p 0.001) groups both significantly improved when compared to placebo.

Studies Evaluating the Effect of Multistrain Probiotics

Four trials examined the impact of multistrain probiotics on IBS patients (Table 5) [28-31]. Different multistrain probiotics were used in the four investigations, although some strains and strain combinations were more common than others.

About 400 participants were involved in a study by Ishaque et al. using a 16-week intervention duration [28]. Patients with moderate to severe IBS symptoms who had subtype IBS-D were included in the study. Their probiotic supplement was supplied as two capsules taken twice daily and contained 14 different bacterial strains. According to the IBS-SSS, which measured symptoms, the probiotic treatment considerably decreased abdominal discomfort in the intervention group (69% from baseline) compared to that in the placebo group (47% from baseline). Regarding the secondary outcomes, it was also discovered that the intervention group significantly improved total IBS symptoms and QoL.

Similar findings were obtained in research by Oh et al. examining the impact of a probiotic capsule given once daily for four weeks to 50 patients with IBS-D [29]. The probiotic supplement (Foodis Lactobacillus) in the capsules consisted of three strains of the *Lactobacillus species*: *L. paracasei, L. salivarius, *and* L. plantarum*. The probiotics group had a substantially greater overall responder rate of improvement of global IBS symptoms indicated by the subject global assessment (SGA) score (80.8%) than the placebo group (45.8%) (p = 0.009). Additionally, the probiotics group had higher overall responder rates as measured by VAS scores (69.2%, 41.7%, p = 0.048).

In a study including 80 IBS patients, Sadrin et al.'s LAPIBSS study sought to show the effectiveness of a two-strain mixture of* Lactobacillus acidophilus* to reduce symptoms of IBS [30]. For eight weeks, the probiotic was consumed twice a day orally. At weeks four and eight (p < 0.0001), both groups' abdominal pain scores significantly improved, but there were no significant differences between the groups at week eight (p = 0.06). The flatus scores at weeks four and eight (p = 0.04) and the composite score at week eight (p = 0.04) revealed significant differences between the groups.

The safety and efficacy of a multistrain probiotic in treating adults with IBS-D were also assessed by Skrzydo-Radomaska et al. [31]. For eight weeks, 48 patients either got a placebo or a probiotic mixture comprising strains of* Lactobacillus, Bifidobacterium, and Streptococcus thermophilus*. After eight weeks of intervention, the probiotic significantly reduced the severity of IBS symptoms in comparison to placebo (IBS-SSS score from baseline: −165.8 ± 78.9, p = 0.005) as well as the severity of pain (p = 0.015) and QoL (p = 0.016). After eight weeks (p = 0.003), the probiotic group demonstrated symptom improvement using the IBS-GIS compared to the placebo group.

Discussion

An increasing body of research, including the biopsychosocial model of IBS, indicates that in individuals with IBS, psychological symptoms (stress, anxiety) may arise as a result of abdominal symptoms (bottom-up); in turn, psychological factors may also influence gut physiology like visceral sensitivity, motility, and stress reactivity. [32]. Through the neurological, endocrine, and immune pathways, it is thought that the gut microbiome and the gut-brain axis both play a significant role in the bidirectional signaling between the brain and the gut, mainly via the first two pathways (neurological and endocrine) (top-down) [33-35]. According to Ng et al. and Yano et al., the gut microbiome synthesizes and modulates neurotransmitters, stimulating the vagus nerve and the enteric nervous system and directly impacting stress reactivity [35,36]. As a result, in IBS cases, the disturbed QOL caused by the cooccurrence of abdominal symptoms, extraintestinal symptoms, and psychiatric symptoms can be improved by reducing IBS-related pain (abdominal symptoms) and regulating the gut microbiome with probiotic therapies [37].

The imbalance in gut microbiota may worsen bloating symptoms because some bacterial species, like *Enterobacteriaceae* and *Clostridia*, are more likely to produce intestinal gas and abnormal patterns of SCFAs than others [38,39]. By reducing intestinal gas production and enhancing gut motility, the alteration of the microbiota ascribed to probiotics may alleviate the symptoms of bloating. Although there is much evidence on this subject, the exact mechanism by which a given species or strain of probiotics works to alleviate IBS symptoms is still hypothetical and must be verified.

Guidelines for probiotic treatment of IBS still need to be determined as of the time of this writing. According to the British Society of Gastroenterology guidelines on managing IBS, probiotics may be an effective treatment for reducing general symptoms and abdominal pain in patients with IBS, updated in 2021 [40]. This finding was in line with the recommendations of the Canadian Association of Gastroenterology and the Japanese Society of Gastroenterology [41,42]. In contrast, the American College of Gastroenterology's recommendations suggest against using probiotics to treat global IBS symptoms [43]. Due to significant heterogeneity, publication bias, inconsistent results in some meta-analyses, several small sample size RCTs without rigorous endpoints based on US Food and Drug Administration (USFDA), and multiple types of probiotics without adequate validations, which may also confound the results, the effectiveness of probiotics in treating patients with IBS has not yet been fully validated [43,44].

In the current evaluation, two studies included participants with only IBS-D [28,31], whereas six studies included participants with all types of IBS [24-30]. The two studies with participants with IBS-D reported a significant improvement in symptoms following probiotic supplementation. The eight studies' eight different interventions ranged from four to 16 weeks. Across all studies, patient symptoms in the interventional arm improved over time at four- and eight-week checkups. This suggests a potential delayed effect of probiotic supplementation in lowering IBS symptoms, which may yield a nonsignificant result in studies that last for a shorter period.

When interpreting the outcomes of this study, several factors need to be considered. First, the evaluation only considers RCTs released during the last five years; hence, it excludes valuable information from earlier works. Second, the results may be impacted by methodological variations in research, such as the probiotic used, the length of the intervention, the sample size, and symptom evaluation. The included study also made use of several symptom evaluation instruments. The validated IBS-SSS was the most commonly utilized questionnaire for symptom evaluation among the eight studies that were included, being used in four of the trials [24,25,28,31]. We recommend consistently using this in future studies based on the reliability of the IBS-SSS.

All of the trials took into account both genders. However, none of them found any appreciable gender differences. According to the findings in the current analysis, there does not appear to be a difference in symptom improvement following probiotic supplementation between male and female individuals. However, there is still a lack of information on how potential gender variations may affect the probiotic therapy of IBS. To identify any possible differences between male and female IBS patients, comprehensive research with separate gender analyses is required in the future.

The probiotic supplements utilized in the trials analyzed in this review exhibit substantial variation in form, dosage, microbial strains, and combinations (Table 4). There was a significant variation in the outcomes between the two research sets due to the division between the ones that used probiotic supplements with one strain and those that used multiple strains. A broad spectrum of multistrain probiotic supplements was administered across the included studies. *Lactobacillaceae and Bifidobacteriaceae* bacterial families, specifically the genera* Lactobacillus *and *Bifidobacterium*, emerged as the prevailing taxa in these probiotic interventions. In prior investigations targeting the fecal microbiota of individuals with IBS, studies reported heightened and diminished* Lactobacillus* counts in IBS patients compared to healthy controls [11,45]. More studies are still needed to validate trends in *Lactobacillus* counts among IBS patients.

Conversely,*Bifidobacterium*was consistently reported to be in reduced quantities in fecal samples of IBS patients in prior studies [5]. This supports the current study's findings, indicating a noteworthy tendency toward symptom improvement in IBS patients who consumed probiotics featuring this bacterial family. However, the precise strains and combinations that yield the most productive results still need to be determined, warranting additional investigative endeavors.

*B. coagulans* is a spore-forming bacteria that is frequently used in commercial probiotic formulations due to its exceptional qualities, including its encapsulated coating, which can protect it from drought conditions and enable it to survive and proliferate in various secretions of the GI tract, including gastric acid, pepsin, pancreatin, digestive enzymes, and bile [46]. Additionally, it can modify the gut flora, boost the body's defenses, and create a variety of proteins, antibacterial agents, and vitamins [47]. Even though there are not many RCTs on the use of various* B. coagulans* strains for IBS patients, it is clear that they are effective and safe. In two distinct studies reviewed in this study, Gupta et al. and Madempudi et al. studied *Bacillus coagulans LBSC (DSM17654)* and *B. coagulans Unique IS2*, respectively [24,26]. According to Gupta et al., *B. coagulans* can dramatically reduce the symptoms of diarrhea and constipation in people with IBS-D and enhance their QoL. Madempudi et al. showed that *B. coagulans Unique IS2* helped reduce IBS-related symptoms in adults with acceptable tolerability, including stomach pain, bloating, urgency, and straining [26]. However, it is essential to remember that the advantages offered by probiotics are strain-specific as opposed to species- and genus-specific. The efficacy of various *B. coagulans* strains in treating IBS was examined in a meta-analysis, which showed that* B. coagulans Unique IS2 *had the highest likelihood of being the best strain for symptom alleviation rate, overall symptom ratings, and the symptom of straining. In contrast, *B. coagulans MTCC5856* took the top spot for reducing bloating and stomach pain [48].

IBS subtypes should be taken into account when treating IBS. Not many studies back up this method, and it can take a few different therapies before the patient starts to feel better. However, there is evidence that specific subtype-specific therapies, including the low-FODMAP diet, are most effective for IBS-D patients, and psyllium husk supplements have the best outcomes for IBS-C [49,50]. Overall, the idea of the dysbiotic gut and the human microbiome as a target for novel therapeutic approaches to alleviate IBS sufferers' GI symptoms indicates the availability of more specialized and customized probiotic supplements in the future.

Limitations

Despite the promising findings, this systematic review has its limitations. Firstly, while efforts were made to conduct a comprehensive literature search in MEDLINE (PubMed) and Google Scholar, some relevant studies may have needed to be included. The inclusion criteria were also restricted to RCTs published within the last five years, potentially excluding older but still pertinent research.

Furthermore, the variability in probiotic formulations across the included studies may introduce a source of heterogeneity. Different strains, doses, and durations of probiotic supplementation were utilized, making it challenging to pinpoint the most effective intervention. Additionally, individual response to probiotics may be influenced by age, gender, diet, and baseline gut microbiota composition, which were not consistently accounted for in the reviewed trials.

Moreover, many studies' reliance on self-reported symptom assessments introduces potential subjective bias. Objective measures or biomarkers of GI function could provide more robust endpoints. Finally, the included trials did not comprehensively address long-term effects and possible adverse events associated with prolonged probiotic use. Therefore, while this review highlights promising trends, caution should be exercised in extrapolating these findings to broader clinical practice, and further research is warranted to address these limitations.

## Conclusions

In conclusion, the systematic review conducted until June 2023 provides compelling evidence for the potential benefits of probiotic supplementation in alleviating IBS symptoms. The analysis encompassed eight recent RCTs; half utilized multistrain probiotics, while the remainder employed monostrain supplements. Remarkably, all trials unanimously reported a significant reduction in IBS symptoms with probiotic treatment compared to a placebo. This consensus among diverse studies using different probiotic formulations underscores the robustness of the findings. While the precise mechanisms by which probiotics exert their beneficial effects remain incompletely understood, the observed alterations in gut microbiota composition suggest a pivotal role in alleviating GI distress.

These findings hold substantial clinical implications for individuals suffering from IBS, offering a potentially effective and accessible intervention to mitigate their symptoms. Nonetheless, it is essential to acknowledge that further research is warranted to refine our understanding of the optimal probiotic strains, dosages, and treatment durations tailored to specific patient profiles. Additionally, long-term studies and investigations into potential side effects are imperative to ensure the safety and efficacy of probiotic interventions. Overall, this systematic review provides a promising foundation for integrating probiotics as a viable adjunctive therapy in managing IBS, emphasizing the need for continued exploration in this field.
